# Biomechanical Stability of Intramedullary Nailing vs Plate Fixation in Displaced Intra-articular Calcaneal Fractures Under Axial Loading: A Systematic Review

**DOI:** 10.1177/24730114261461284

**Published:** 2026-07-24

**Authors:** Ivana Djurica, Martin Pompach, Tim Schepers

**Affiliations:** 1 Trauma Unit, Department of Surgery, Amsterdam UMC location Meibergdreef, Amsterdam, the Netherlands; 2 Department of Traumatology, Regional Hospital Pardubice, Czech Republic

**Keywords:** Calcaneal fracture, intra-articular, intramedullary nail, C-Nail, Calcanail, biomechanics, cadaver study, FEA

## Abstract

**Background:**

From a biomechanical perspective, intramedullary fixation offers potential benefits, including a central load-sharing axis that may better withstand axial and bending forces, while preserving the periosteal blood supply and reducing soft tissue damage. This review aimed to determine whether intramedullary nailing in displaced intra-articular fractures provides comparable or superior biomechanical stability to plate fixation.

**Methods:**

A literature search identified studies comparing the biomechanical stability of intramedullary fixation with plate fixation, screw-only fixation, or combined constructs in calcaneal fractures. Eligible studies used human cadaveric specimens or computational models, examined biomechanical outcomes such as stiffness, load to failure, displacement, or stress distribution, and employed laboratory, cadaveric (n = 3), or finite element analyses (n = 4).

**Results:**

Experimental and numerical studies showed that intramedullary fixation consistently provides greater or comparable construct stiffness and load to failure than locking plates, with no implant failures observed. Plates produced higher stress concentrations at screw junctions, whereas intramedullary nails yielded a more uniform stress distribution in most models; however, 1 finite element analysis study reported higher calcaneal bone stress with intramedullary nail fixation.

**Conclusion:**

Intramedullary calcaneal fixation provides comparable or superior biomechanical stability to plate fixation, especially under cyclic axial loading. Construct durability appears more dependent on axial load sharing than on implant category alone. Whether these biomechanical findings translate to improved clinical outcomes remains to be demonstrated.

## Introduction

Only about 2% of all fractures involve the calcaneus, and of these, displaced intra-articular fractures account for between 60% and 75%.^1,2^

Although not new, recent intramedullary (IM) nails have been developed specifically for calcaneal fractures, particularly displaced intra-articular fractures (DIACFs) such as Sanders type II and III.^3^ Examples include the C-Nail (Medin), an IM nail that is inserted via a posterior incision to stabilize the calcaneus with 6 interlocking screws, and the Calcanail (FH Ortho), which uses a posterior working channel with 2 locking screws.^4^

Clinically, Goldzak et al^5^ first reported early experience with the Calcanail, focusing on feasibility, perioperative outcomes, and complications. The first PubMed-indexed clinical outcome study of the C-Nail was published by Zwipp et al,^6^ who reported satisfactory mid-term outcomes and complication profiles, supporting its use as an alternative fixation method to plate or screw fixation in selected cases.

Clinical studies suggest that these intramedullary systems may maintain reduction comparable to lateral plate fixation with fewer wound complications. In a matched-pair analysis, Herlyn et al^7^ reported a complication rate of 5% in patients treated with IM nails compared with 50% in those treated with plates. Similarly, Zeman et al^8^ found comparable outcomes between IM nailing and plate fixation, but noted fewer complications, such as skin necrosis and deep infection, in the IM nail group. However, whether these findings are supported in construct stability compared with alternative fixation methods remains unclear.

Biomechanically, intramedullary fixation offers theoretical advantages, including a central load-sharing axis that may better resist axial and bending stresses while minimizing soft tissue disruption.^4,9^ Conversely, plate osteosynthesis provides multi-planar buttressing and direct control of the lateral wall, potentially conferring superior resistance to rotational displacement in complex fracture patterns.^10^ Greater rigidity may allow earlier weight-bearing, with benefits such as earlier return to work and less muscle atrophy.^11,12^

This review aims to assess the current literature and determine whether IM nailing provides biomechanical stability equivalent to or superior to that of plate fixation in DIACFs.

## Methods

### Study Design and Reporting Standards

This systematic review was conducted in accordance with the Preferred Reporting Items for Systematic Reviews and Meta-Analyses (PRISMA) guidelines, using predefined eligibility criteria and a methodologic approach.

### Literature Search Strategy

A literature search was performed to identify studies evaluating the biomechanical stability of intramedullary fixation compared with plate fixation, screw-only fixation, or combined constructs in calcaneal fractures.

Electronic searches were conducted in PubMed (MEDLINE) and Embase. The initial PubMed scoping search on 11 November 2025 yielded 11 records. An additional search on 11 January 2026 yielded 13 records. Afterwards, the search was expanded to Embase to maximize coverage of biomechanical and experimental studies; there were no additional studies found.

No restrictions were applied on language or publication status. Reference lists of all included studies and relevant reviews were manually screened for additional eligible studies.

The PubMed search strategy included terms such as *calcaneus*, *biomechanical*, *finite element*, *cadaver*, *intramedullary nail*, *interlocking nail*, *plate*, *screw-only*, and their variations. A comparable search strategy was adapted for Embase.

### Eligibility Criteria

Studies were considered eligible if they met the following criteria:

- Population: Human cadaveric specimens or validated computational models simulating calcaneal fractures
- Intervention: Intramedullary fixation of calcaneal fractures
- Comparator: Plate fixation, screw-only fixation, or combined fixation constructs
- Outcomes: Biomechanical outcomes, including but not limited to construct stiffness, load to failure, displacement, micromotion, or stress distribution
- Study design: Biomechanical laboratory studies, cadaveric studies, and finite element analyses (FEAs)

Clinical outcome studies, case reports, narrative reviews, editorials, conference abstracts without full data, and purely surgical technique descriptions without biomechanical testing were excluded.

### Study Selection

All records were imported into reference management software, and duplicates were removed as needed. Two reviewers independently screened titles and abstracts for eligibility. Full-text articles were assessed for inclusion based on the predefined criteria. Disagreements were resolved through discussion and consultation with a third reviewer.

### Data Extraction

Data were extracted independently by 2 reviewers using a standardized form. Extracted variables included the following:

- Study characteristics (author, year, country)
- Study type (cadaveric, finite element [FE], in vitro)
- Fracture model and classification
- Fixation constructs evaluated
- Loading conditions and testing protocols
- Primary biomechanical outcomes
- Key findings and conclusions

### Methodologic Quality and Risk-of-Bias Assessment

Methodologic quality was assessed using a customized appraisal framework adapted from published tools for experimental and biomechanical research. Domains assessed included specimen preparation, fracture reproducibility, fixation standardization, loading protocol validity, and outcome reporting.^13^ There was no formal certainty assessment done.

### Data Synthesis

Because of anticipated heterogeneity in fracture models, fixation constructs, and testing protocols, a qualitative narrative synthesis was planned. Where outcome measures were sufficiently comparable, results were summarized descriptively in tables. Meta-analysis was not planned a priori. Formal assessment of reporting bias was not performed because of the limited number and heterogeneity of studies. LLM-model GPT-4.1 was used to screen for grammatical inconsistencies and improve clarity in the manuscript. All changes were manually reviewed and approved before applying.

## Results

### Article Selection

**Figure 1.**
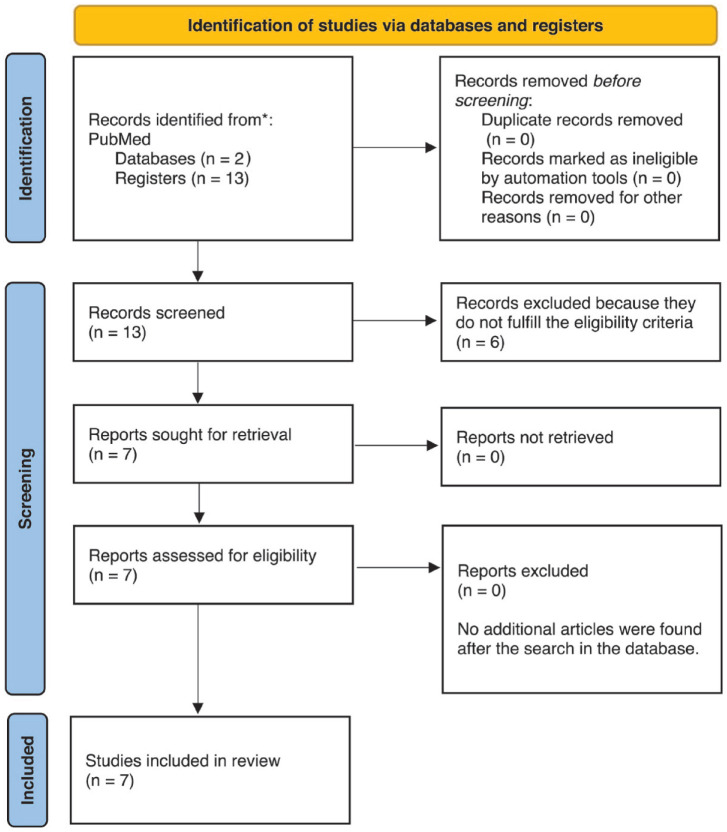
PRISMA flow chart of literature search.

The search identified 13 records; there were no duplicates, 7 full-text articles were assessed, and 7 studies were included (Figure 1). Studies without method comparison or focusing solely on plate or screw fixation were excluded. Additionally, no additional articles were found on Embase and Google Scholar using terms such as *calcaneal fracture*, *IM nail*, *plates*, *screw-only*, *biomechanical*, and *osteosynthesis outcomes*. Given the experimental nature of the included studies, certainty of evidence was expected was expected to be low and was categorized qualitatively.

### Article Overview

Cadaveric studies (n = 3) and FEA (n = 4) evaluated Sanders type II and type III fracture patterns, comparing calcaneus-specific intramedullary nails with (locking) plate constructs. Loading conditions varied: FEA models predominantly applied axial loading, whereas 2 cadaveric studies applied axial loading in combination with cyclic protocols. An overview of study characteristics, fracture models, fixation strategies, and test protocols is provided in Table 1.

**Table 1.** Study characteristics and experimental models.

First Author (Year)	Journal	Study Type	Model/Specimen	Fracture Model	Intramedullary Fixation	Comparator	Test Protocol	Primary Outcomes
Goldzak^14^ (2014)	*Injury*	Cadaveric	Dry human calcanei	Sanders IIB	Calcanail	Angular stable plate	Axial compression to failure	Stiffness, LTF, displacement
Reinhardt^15^ (2016)	*Foot & Ankle Int.*	Cadaveric	Fresh frozen hindfoot	Sanders IIB	C-Nail, Calcanail	Interlocking plate	Cyclic + LTF	Stiffness, LTF, IFM, Böhler angle
Stefanov^16^ (2022)	*Injury*	Cadaveric study (comparative)	*Model*: 9 paired fresh frozen human cadaveric lower legs (n = 18 calcanei) Mean age 73 y	Sanders III AB posterior facet fracture, Kinner II B CCJ fracture, lateral wall comminution and posterior tuberosity defect	C-Nail	VA locking lateral calcaneal plate and VA locking anterolateral calcaneal plate + longitudinal screw	Axial cyclic loading to failure (progressively increasing load), motion tracking + radiography	Stiffness, load/cycles to failure, varus deformation, plantar gapping, CCJ displacement, Böhler angle loss
Ni^17^ (2019)	*Med Eng Phys*	FE	CT-based FE Model: 62 y, 70 kg, 170 cm, gender unknown	Sanders IIIAB	Modified Calcanail and standard Calcanail	Plate	Axial load 700 N	Stiffness, micromotion, stress
Pînzaru^18^ (2023)	*J Pers Med*	FE	CT-based FE *Model*: female, 30 y, 70 kg, 175 cm	Sanders IIB	C-Nail	Locking plate	Axial load 350-700 N	Stress, displacement
Wu^19^ (2024)	*BMC Musculoskelet Disord*	FE + experiment	CT FE + compression test *Model*: 36 y, 70 kg, height and gender unknown	Sanders II to III	IM calcaneal nail	Locking plate	Axial compression	Stiffness, stress, displacement
Han^20^ (2026)	*Nature*	FE + clinical follow-up	CT-based FE model + 23 clinical cases Model: 31 y, 71 kg, 176 cm, gender unknown	Sanders II to III, III for model	IM nail model	LCP + MIP + percutaneous ST screw	Axial loading, stress loading in FE; clinical postoperative follow-up	Von Mises stress (bone/implant), fragment displacement; clinical outcomes: AOFAS/MFS/VAS

Abbreviations: AOFAS, American Orthopaedic Foot & Ankle Society ankle-hindfoot scale; CCJ, calcaneocuboid joint; FE, finite element; IFM, interfragmentary motion; IM, intramedullary; LCP, locking compression plates; LTF, Load to failure; MFS, Maryland Foot Score; MIP, minimally invasive plates; ST, sustentaculum tali; VA, variable-angle; VAS, visual analog scale.

### Study Design and Biomechanical Assessment

The included studies were heterogeneous, comprising cadaveric and FEA with varying fracture types, fixation constructs, and loading conditions. Methodological limitations mainly related to model assumptions rather than systematic bias. An overview of study designs, testing frameworks, and assessment methods is provided in Table 2.

**Table 2.** Study design and biomechanical assessment frameworks.

First Author (Year)	Notes	Origin of the Assessment Method
Goldzak^14^ (2014)	Axial compression to failure; stiffness + displacement metrics	Bardet et al^21^
Reinhardt^15^ (2016)	Cyclic + LTF with subtalar fragment stability assessment	Redfern et al and Zech et al^22,23^
Stefanov^16^ (2022)	Progressive cyclic axial loading to failure; stiffness + 3D displacement (varus, plantar gapping, CCJ) + Böhler angle.	Cyclic axial loading with motion tracking model adapted from prior calcaneal biomechanical studies (e.g., Rausch; Reinhardt).^14,24^
Ni^17^ (2019)	Discrepancy between numerical reference and text written	Smerek et al^25^
Pînzaru^18^ (2023)	FE comparison: plate shows higher stress concentrations	FE methodology per study (Ansys/SpaceClaim workflow described in article)
Wu^19^ (2024)	FE + experimental compression test; compares nail vs plate	Self-developed / combined FE–experimental framework
Han^20^ (2026)	FE comparison of 4 constructs + clinical follow-up; reports stress and fragment displacement (threshold referenced)	FE models were developed based on previously established methods and applied for comparative construct analysis.

Abbreviations: CCJ, calcaneocuboid joint; FE, finite element; LTF, load to failure.

### Construct Stiffness

Intramedullary fixation consistently demonstrated superior construct stiffness compared with locking plate fixation across studies. In paired cadaveric testing, IM nail constructs showed about 3 times greater stiffness than angular-stable plate constructs, indicating improved resistance to axial deformation.^14^ In another cadaveric study, the IM construct showed the highest stiffness (305.5 N/mm), compared with VA-LP (143.8 N/mm) and VA-LP with longitudinal screws (247.1 N/mm), although with the numbers available, no significant difference could be detected.^16^ FEA corroborated these findings; the modified Calcanail with transfixion screw achieved the highest stiffness (552 N/mm), followed by the standard Calcanail (522 N/mm) and plate fixation (454 N/mm) under a 700 N vertical load.^17^ Dynamic mechanical testing further revealed variability among fixation systems, with the Calcanail showing the highest construct stiffness, followed by the Rimbus locking plate and the C-Nail.^15^ Numerical models of Sanders type II and III fractures showed slightly greater overall stiffness in IM nailing than plates.^19^ A quantitative comparison of reported construct stiffness across studies is summarized in Table 3.

**Table 3.** Summary of Biomechanical Findings.

First Author (Year)	Stiffness (Nail vs Plate)	Load to Failure	Interfragmentary Motion	Stress Distribution	Key Finding
Goldzak^14^ (2014)	Nail >> plate	Higher with nail	Lower subtalar displacement	—	IM nail showed superior primary stability
Reinhardt^15^ (2016)	Comparable	No significant difference	Comparable; stable subtalar fragment	—	C-Nail had fewer cyclic failures
Stefanov^16^ (2022)	Nail > LP + longitudinal screw > LP	Nail ≈ LP + longitudinal screw > LP	Less varus + better Böhler angle maintenance with nail and LP + longitudinal screw	—	Constructs with longitudinal strut (nail or LP + longitudinal screw) showed superior stability vs LP alone
Ni^17^ (2019)	Highest with modified nail	—	Lowest with modified nail	Lower with nail	Transfixation screw improved stability in the modified Calcanail
Pînzaru^18^ (2023)	Slightly higher with nail	—	Lower with nail	Plate showed higher stress	Nail had more favorable stress distribution
Wu^19^ (2024)	Slightly higher with nail	Plate higher at extreme load	Less block displacement with nail	Lower bone stress with nail	Both stable; IM nail favored load distribution
Han^20^ (2026)	—	—	Fragment displacement in all constructs below threshold (reported as <150 μm)	Peak calcaneus stress highest in IM nail model; implant stress highest in LCP model	Percutaneous ST screw construct showed biomechanical stability comparable to other constructs in FEA; IM nail model did not show lower bone stress in this study

Abbreviations: FE, finite element; IM, intramedullary; LCP, locking compression plates; LP, locking plate.

### Load to Failure

Intramedullary fixation generally achieved higher or comparable load-to-failure values relative to plate fixation. In paired mechanical testing, IM nail constructs sustained markedly higher loads before failure than plate constructs.^14^ In a 3-group comparison of C-Nail, Calcanail, and Rimbus locking plate fixation, the C-Nail demonstrated the highest mean load to failure, although differences between groups did not reach statistical significance. The Calcanail failed 73.5% more frequently than the C-Nail, while the Rimbus locking plate had a 52% higher failure rate.^15^ Both the VA-LP with longitudinal screws and the C-Nail withstood higher loads and exhibited varus failure later than the VA-LP only.^16^ In contrast, FEA demonstrated that the intramedullary fixation construct reached its failure threshold at a load approximately 2400 N lower than that of the LP fixation group.^19^ Bone mineral density was comparable across groups and did not correlate with load to failure, indicating that construct strength was predominantly influenced by fixation. Reported load-to-failure outcomes and testing conditions are summarized in Table 3.

### Failure Modes

No implant breakage was observed across studies. Failure patterns were consistently localized to the anterior of the calcaneus, regardless of the fixation method.^14^ Dynamic testing revealed a notably lower failure rate for the C-Nail compared with the Calcanail, suggesting superior fatigue resistance rather than differences in ultimate strength as the primary differentiator between nail designs.^15^ In Stefanov et al,^16^ failure consistently occurred at the angle of Gissane, with posterior facet displacement generally remaining ≤2 mm across groups. However, anterior process screw cut-through was common, and inferior nail pull-out occurred in all C-Nail specimens.

### Stress Distribution

FEA demonstrated clear differences in stress distribution between fixation strategies. Plate fixation consistently generated higher stresses within the implant and adjacent cortical bone, particularly at plate-screw junctions, whereas IM nail systems exhibited more uniform stress transfer through the implant and surrounding bone.^17^ All predicted implant stresses remained well below the ultimate strength of titanium alloys, indicating a sufficient mechanical safety margin. Under axial loading conditions of 350 N (partial weight bearing) and 700 N (full weight bearing), additional numerical modeling confirmed that intramedullary fixation resulted in lower bone stress and reduced fragment displacement compared with plate fixation under axial loading (110 and 360 MPa, respectively). The observed stress distribution of the plate was uneven compared with the IM.^18^ Additional FE modeling showed that intramedullary fixation concentrates stress within the implant while reducing stress transferred to bone compared with locking plate fixation, whereas plating spreads implant stress across the plate-screw system but is associated with higher bone stress, particularly in Sanders type II fractures.^19^ On the other hand, a recent FEA showed that the IM nail generated the highest stress in the calcaneal bone, the locking plate experienced the highest implant stress, and the ST screw fixation produced the lowest stresses in both bone and implant.^20^ Differences in stress distribution and fragment loading are summarized in Table 3.

### Fragment Motion and Joint Stability

Subtalar joint displacement under low axial load was consistently lower with IM nail fixation, particularly at posterior measurement sites, reflecting improved joint stability.^17^ FE models predicted fracture micromotion of ≤0.1 mm for all fixation methods, a range considered favorable for fracture healing.^17^ Although changes in Böhler angle and interfragmentary motion did not differ significantly between fixation types in some experimental studies, nail-based constructs consistently demonstrated reduced motion at central and subtalar fragments.^15^ Similarly, Han et al^20^ reported fragment motion less than 150 μm across all conditions, and Wu et al^19^ observed slightly lower block displacement with IM nail fixation. These findings parallel cadaveric biomechanical data demonstrating greater varus deformation and Böhler angle reduction in LP constructs compared with fixation strategies that incorporate intramedullary or longitudinal support.^16^

## Discussion

Current evidence indicates that intramedullary fixation for intra-articular calcaneal fractures provides biomechanical performance comparable to, and in some respects better than, plate- and screw-based fixation. Across both computational and cadaveric models investigations, intramedullary constructs generally demonstrated higher or equivalent stiffness, reduced displacement under load, and more favorable load-sharing behavior.^19^

Although biomechanical models frequently demonstrate higher construct stiffness for intramedullary devices, clinical series document variable maintenance of reduction. For example, Pompach et al^26^ reported a 3.5° decline in the Böhler angle from immediate postoperative values to 12 months, consistent with postoperative settling. However, in a comparative cohort focused on correction loss, Gajdošíková et al^27^ defined radiographic failure as a Böhler angle decrease of more than 5° and/or height loss, reporting this in approximately 42% of nail-treated cases. Conversely, some comparative studies report no measurable loss of reduction during follow-up.^28,29^ Stefanov et al^16^ further refined this interpretation by showing that constructs incorporating a longitudinal strut, either an intramedullary nail or an anterolateral plate augmented with a longitudinal screw, better resisted progressive varus than isolated lateral plating. Although initial stiffness differences were not statistically significant with the numbers available, this indicates that stiffness alone does not predict durability under cyclic loading. Axial load sharing along the calcaneal mechanical axis and implant configuration appear more important for preventing angular collapse than implant category.^20^

FE studies consistently showed that intramedullary systems transferred more load to the implant, resulting in lower, more evenly distributed stress. An important exception was reported by Han et al,^20^ who found that IM nail fixation generated the highest bone stresses, whereas screw-only fixation demonstrated the most favorable stress distribution. This highlights that stress transfer behavior may depend on construct design rather than being inherent to intramedullary fixation. As the only study to directly compare IM nail with screw-only constructs, further evaluation is warranted before broader conclusions can be drawn.

Several limitations should be acknowledged. Although favorable clinical outcomes have been reported for the intramedullary fixation, the biomechanical basis remains incompletely defined. Direct biomechanical validation of stability relative to plate- or screw-only fixation is limited, and the available experimental and finite-element studies are few and heterogeneous with respect to study design, fracture simulation, and implant configuration. In addition, the absence of standardized biomechanical outcome metrics and reporting thresholds limits direct quantitative comparison across studies. This heterogeneity restricts meaningful comparison across studies and limits the strength of mechanistic conclusions. FEAs, although valuable for exploring mechanical behavior, depend on assumptions regarding material properties, boundary conditions, and fracture representation that may not accurately reflect in vivo conditions. Additionally, the use of the AOFAS score in Han et al^20^ limits interpretation, as this measure lacks validity and is no longer recommended as a primary outcome. Also, biomechanical superiority does not necessarily translate into improved clinical outcomes, as factors such as soft tissue preservation, surgical approach, and complication rates remain critical determinants of success. Finally, there was no formal assessment of the certainty of the evidence.

## Conclusion

The available biomechanical and numerical evidence suggests that intramedullary calcaneal fixation offers comparable or superior stiffness, reduced displacement, and generally more favorable stress distribution compared with plate fixation, particularly under cyclic loading, supporting a stable strategy for DIACFs. Overall biomechanical performance is comparable; durability depends more on load sharing than implant type. Further standardized biomechanical testing and clinical research are necessary to substantiate long-term clinical advantages, as biomechanical stability alone does not necessarily translate into improved clinical outcomes.

## Supplemental Material

sj-pdf-1-fao-10.1177_24730114261461284 – Supplemental material for Biomechanical Stability of Intramedullary Nailing vs Plate Fixation in Displaced Intra-articular Calcaneal Fractures Under Axial Loading: A Systematic Review Supplemental material, sj-pdf-1-fao-10.1177_24730114261461284 for Biomechanical Stability of Intramedullary Nailing vs Plate Fixation in Displaced Intra-articular Calcaneal Fractures Under Axial Loading: A Systematic Review by Ivana Djurica, Martin Pompach and Tim Schepers in Foot & Ankle Orthopaedics
