# Supplementary material for: Biomechanical Stability of Intramedullary Nailing vs Plate Fixation in Displaced Intra-articular Calcaneal Fractures Under Axial Loading: A Systematic Review
Source: Foot Ankle Orthop. 2026 Jul 24;11(3):24730114261461284. doi: 10.1177/24730114261461284 (PMC13400927; doi:10.1177/24730114261461284)
Supplement: sj-pdf-1-fao-10.1177_24730114261461284 – Supplemental material for Biomechanical Stability of Intramedullary Nailing vs Plate Fixation in Displaced Intra-articular Calcaneal Fractures Under Axial Loading: A Systematic Review [file sj-pdf-1-fao-10.1177_24730114261461284.pdf]

Conflict of Interest (COI) Disclosure Form FAI / FAO

Purpose:
All authors submitting a manuscript to this peer-reviewed journal **must disclose any conflicts of interest (COI) related to the submitted paper**.
No other disclosures are required or desired.

A conflict of interest typically involves **financial relationships (most commonly payments to surgeons or authors)** that could reasonably be perceived as influencing the submitted work, whether **received in the past or anticipated in the future**.

**Author Name:**	**Ivana Djurica**
**Manuscript Title:**	IS INTRAMEDULLARY NAILING IN DISPLACED CALCANEAL FRACTURES MORE STABLE THAN SCREWS OR PLATES? A BIOMECHANICAL REVIEW.
**Date:**	**10-03-2026**

Conflict of Interest Disclosure:

Do you or an immediate family member (spouse, domestic partner, or dependent child) have financial conflicts of interest valued over $500 related to this manuscript or its subject matter?
(Examples include consulting fees, honoraria, royalties, stock or stock options, research funding, paid advisory roles, product or material support, or intellectual property interests (e.g., potential future royalties) valued over $500, received within the past three years or expected in the future.)

☒ **No — I have no conflicts of interest to disclose.**

☐ **Yes — I have a conflict of interest to disclose.**
If **Yes**, please briefly describe the financial relationship(s) below, including the company/entity involved and the nature of the payment or support:

Click or tap here to enter text.

Updates: If a relevant conflict of interest arises or changes after submission and before publication, an updated disclosure must be provided.

Publication of Disclosure: All disclosed conflicts of interest will be published with the article and completed forms will be made available as an online supplement.

Conflict of Interest (COI) Disclosure Form FAI / FAO

Purpose:
All authors submitting a manuscript to this peer-reviewed journal **must disclose any conflicts of interest (COI) related to the submitted paper**.
No other disclosures are required or desired.

A conflict of interest typically involves **financial relationships (most commonly payments to surgeons or authors)** that could reasonably be perceived as influencing the submitted work, whether **received in the past or anticipated in the future**.

**Author Name:**	**Dr. Martin Pompach**
**Manuscript Title:**	IS INTRAMEDULLARY NAILING IN DISPLACED CALCANEAL FRACTURES MORE STABLE THAN SCREWS OR PLATES? A BIOMECHANICAL REVIEW.
**Date:**	**10-03-2026**

Conflict of Interest Disclosure:

Do you or an immediate family member (spouse, domestic partner, or dependent child) have financial conflicts of interest valued over $500 related to this manuscript or its subject matter?
(Examples include consulting fees, honoraria, royalties, stock or stock options, research funding, paid advisory roles, product or material support, or intellectual property interests (e.g., potential future royalties) valued over $500, received within the past three years or expected in the future.)

☒ **No — I have no conflicts of interest to disclose.**

☐ **Yes — I have a conflict of interest to disclose.**
If **Yes**, please briefly describe the financial relationship(s) below, including the company/entity involved and the nature of the payment or support:

Click or tap here to enter text.

Updates: If a relevant conflict of interest arises or changes after submission and before publication, an updated disclosure must be provided.

Publication of Disclosure: All disclosed conflicts of interest will be published with the article and completed forms will be made available as an online supplement.

Conflict of Interest (COI) Disclosure Form FAI / FAO

Purpose:
All authors submitting a manuscript to this peer-reviewed journal **must disclose any conflicts of interest (COI) related to the submitted paper**.
No other disclosures are required or desired.

A conflict of interest typically involves **financial relationships (most commonly payments to surgeons or authors)** that could reasonably be perceived as influencing the submitted work, whether **received in the past or anticipated in the future**.

**Author Name:**	**Dr. Tim Schepers**
**Manuscript Title:**	IS INTRAMEDULLARY NAILING IN DISPLACED CALCANEAL FRACTURES MORE STABLE THAN SCREWS OR PLATES? A BIOMECHANICAL REVIEW.
**Date:**	**10-03-2026**

Conflict of Interest Disclosure:

Do you or an immediate family member (spouse, domestic partner, or dependent child) have financial conflicts of interest valued over $500 related to this manuscript or its subject matter?
(Examples include consulting fees, honoraria, royalties, stock or stock options, research funding, paid advisory roles, product or material support, or intellectual property interests (e.g., potential future royalties) valued over $500, received within the past three years or expected in the future.)

☒ **No — I have no conflicts of interest to disclose.**

☐ **Yes — I have a conflict of interest to disclose.**
If **Yes**, please briefly describe the financial relationship(s) below, including the company/entity involved and the nature of the payment or support:

Click or tap here to enter text.

Updates: If a relevant conflict of interest arises or changes after submission and before publication, an updated disclosure must be provided.

Publication of Disclosure: All disclosed conflicts of interest will be published with the article and completed forms will be made available as an online supplement.
